# *Streptococcus pyogenes* c-di-AMP Phosphodiesterase, GdpP, Influences SpeB Processing and Virulence

**DOI:** 10.1371/journal.pone.0069425

**Published:** 2013-07-15

**Authors:** Kyu Hong Cho, Song Ok Kang

**Affiliations:** 1 Department of Microbiology, Southern Illinois University, Carbondale, Illinois, United States of America; 2 Department of Physiology, School of Medicine, Southern Illinois University, Carbondale, Illinois, United States of America; Montana State University, United States of America

## Abstract

Small cyclic nucleotide derivatives are employed as second messengers by both prokaryotes and eukaryotes to regulate diverse cellular processes responding to various signals. In bacteria, c-di-AMP has been discovered most recently, and some Gram-positive pathogens including *S. pyogenes* use this cyclic nucleotide derivative as a second messenger instead of c-di-GMP, a well-studied important bacterial second messenger. GdpP, c-di-AMP phosphodiesterase, is responsible for degrading c-di-AMP inside cells, and the cellular role of GdpP in *S. pyogenes* has not been examined yet. To test the cellular role of GdpP, we created a strain with a nonpolar inframe deletion of the *gdpP* gene, and examined the properties of the strain including virulence. From this study, we demonstrated that GdpP influences the biogenesis of SpeB, the major secreted cysteine protease, at a post-translational level, susceptibility to the beta lactam antibiotic ampicillin, and is necessary for full virulence in a murine subcutaneous infection model.

## Introduction

**Figure 1:**
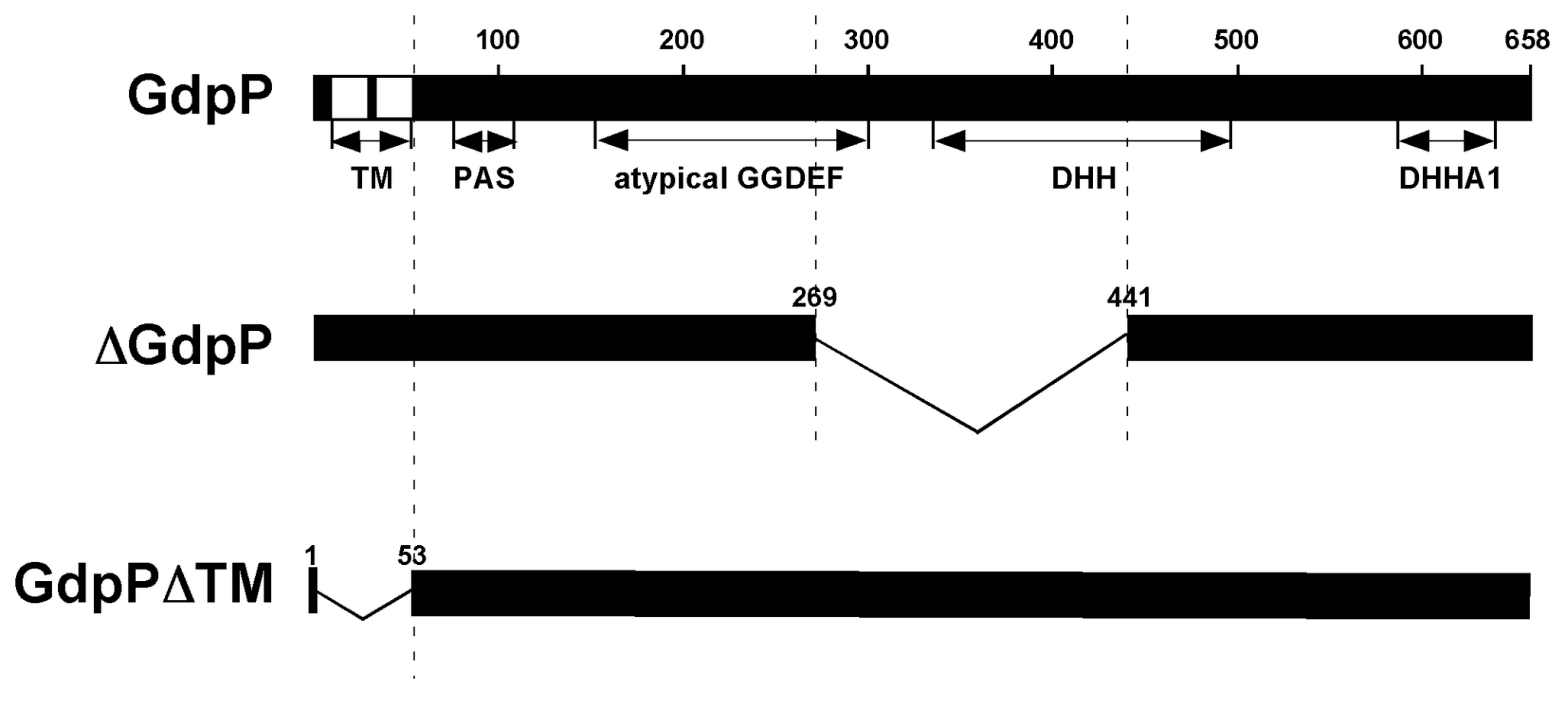
Domain structure of the GdpP protein. The N-terminus of GdpP contains 2 transmembrane helices (TM, white boxes, residues 10-52), a degenerated PAS domain (residues 76-108), an atypical GGDEF domain (residues 150-298), a DHH domain (residues 335-496) and a DHHA1 domain (residues 587-639). GdpP mutant proteins expressed as recombinant proteins in this study are shown below the original wild type protein. ∆GdpP, nonpolar inframe GdpP deletion; and GdpP∆TM, transmembrane domain-deleted GdpP.

In bacteria, cyclic dimeric guanosine 3’,5’-monophosphate (c-di-GMP) has recently emerged as an important second messenger regulating gene expression [1]. In human pathogenic bacteria such as *Pseudomonas aeruginosa*, c-di-GMP has an important role in regulating virulence factor expression and biofilm formation [2]. However, no evidence has been shown yet that some important Gram-positive pathogens including *Staphylococcus aureus*, *Streptococcus pyogenes*, and *Listeria monocytogenes* synthesize c-di-GMP. Furthermore, *Staphylococcus* and *Streptococcus* do not even possess enzymes with domains responsible for c-di-GMP synthesis (a typical GGDEF diguanylate cyclase domain) or hydrolysis (an EAL or HD-GYP phosphodiesterase domain) [3,4]. Recent studies, however, showed that these Gram-positive pathogens synthesize a similar cyclic nucleotide, cyclic dimeric adenosine 3’, 5’-monophosphate (c-di-AMP) instead [5–7].

c-di-AMP is synthesized by a protein DacA, which contains a di-adenylate cyclase domain (DAC domain) [5–8]. The production of c-di-AMP seems to be essential for viability because the DAC domain protein cannot be deleted using traditional genetic techniques in bacteria such as *Staph* *aureus* [5], *Mycoplasma genitalium* [9], *Mycobacterium* *pulmonis* [10], *Streptococcs* *pneumoniae* [11], and *L. monocytogenes* [7] that possess only one DAC protein. Our model pathogenic bacterium, *S. pyogenes*, also has one DAC protein (Spy_1036). The c-di-AMP synthesized by the DacA protein is degraded by a membrane-anchored intracellular protein GdpP [5]. GdpP has a non-cleavable signal sequence followed by two N-terminal transmembrane helixes, a highly degenerated PAS sensory domain, an atypical GGDEF domain, and a DHH/DHHA1 domain [4,5] (Figure 1). The DHH/DHHA1 domain shows c-di-adenylate hydrolase activity both *in vivo* and *in vitro* [4,5]. As expected, the deletion of the *gdpP* gene increases the concentration of c-di-AMP more than 10 fold in *Staph* *aureus* [5] and over 4 fold in early sporulating *B. subtilis* [12]. GdpP appears to be involved in cell wall homeostasis/cell division and stress response in bacteria [4,5,13]. In *Staph* *aureus* , *gdpP* deletion not only suppresses the growth defect caused by stress on envelope associated with an inability to synthesize lipoteichoic acid, but also increases the degree of peptidoglycan cross-linking, decreases susceptibility to cell wall targeting antibiotics, and reduces cell size by ~ 20% [5]. In *B. subtilis*, the deletion of the *gdpP* ortholog, *yybT*, increases sporulation efficiency of cells challenged with the DNA gyrase inhibitor nalidixic acid, and decreases the susceptibility to beta-lactam antibiotics [4,13]. However, the role of GdpP in the physiology and virulence of *S. pyogenes* has not yet been elucidated.

*S. pyogenes* is a strict human pathogen that causes diseases ranging from mild infections such as strep throat and impetigo to life threatening diseases like cellulitis, necrotizing fasciitis, rheumatic heart disease, and toxic shock syndrome. *S. pyogenes* secrets a multitude of virulence factors, and one of the well-studied streptococcal virulence factors is SpeB (secreted pyrogenic exotoxin B). SpeB is a cysteine protease encoded on a chromosomal gene [14], so most, if not all, streptococcal strains produce SpeB, and the secreted amount of SpeB of some strains is more than 90% of the total secreted proteins at the stationary phase [15]. Potential targets of SpeB in infection were identified from many studies. SpeB damages host tissue directly by destroying human matrices such as fibronectin and vitronectin [16], or indirectly by activating host matrix metalloproteases whose expression is tightly restricted in the host [17]. SpeB has also been shown to convert biologically inactive host proteins to biologically active forms that have immunomodulating activities: SpeB can convert interleukin 1beta precursor to active IL-1beta that is a strong inflammatory mediator [18], and kininogen to active kinins such as bradykinin that is a proinflammatory and vasoactive peptide hormone [19]. SpeB releases some biologically active streptococcal virulence factors which are normally associated on the cell surface such as M protein, protein F, and C5a peptidase [20]. Liberation of those surface proteins has been shown to disturb the host immune system [20]. Through these characteristics, SpeB significantly contributes to the deadly disease, necrotizing fasciitis (For a review, see 21).

SpeB production is regulated transcriptionally and post transcriptionally. Initially, SpeB is translated as a preproprotein (43 kDa) containing a signal sequence and a pro region of SpeB. When SpeB is secreted by the Sec system, the signal sequence is cleaved off to form the proSpeB zymogen (~40 kDa), which is still an inactive protease. After being secreted, the zymogen is folded and processed to the active protease (23 kDa) by multiple cleavages in the pro region. Previous studies showed that this processing is influenced by peptidyl-prolyl *cis-*trans isomerases [22–24], and HtrA, a cell surface protease [25,26].

In this study, to investigate the role of GdpP in the physiology and virulence of *S. pyogenes*, we deleted the *gdpP* gene (*spy_2184*) and examined the deletion effect. Here, we provide the evidence that the c-di-AMP phosphodiesterase GdpP is required for the processing of SpeB and full virulence of *S. pyogenes* in an animal infection model.

## Materials and Methods

### Bacterial strains and Media

*S. pyogenes* HSC5 [27] was used for most experiments and strain construction. To culture *S. pyogenes* in liquid, two media, THY and *C* *medium* , were used. THY medium, Todd-Hewitt medium (BBL) supplemented with 0.2% yeast extract (Difco), was used for routine culture, and *C* *medium* was exclusively employed to determine SpeB expression [23]. Liquid culture was set at 37°C in sealed tubes without shaking. For solid media, Bacto agar (Difco) was added to a final concentration of 1.4% (w/v). Cultures on solid media were incubated under anaerobic conditions (<0.03% O_2_ and 15% CO_2_) employing a commercial gas generator (GasPak EZ Anaerobe container system, catalogue no. 260678, BBL). For plasmid construction, *Escherichia coli* DH5α [28] or TOP10 (Invitrogen), which was cultured in Luria-Bertani broth, was employed. When appropriate, antibiotics were added to the media at the following concentrations unless specified; ampicillin 100 µg/ml for *E. coli*; kanamycin 50 µg/ml for *E. coli* and 500 µg/ml for *S. pyogenes*; erythromycin 500 µg/ml for *E. coli* and 1 µg/ml for *S. pyogenes*; chloramphenicol 7 µg/ml for *E. coli* and 3 µg/ml for *S. pyogenes*.

### Manipulation of DNA

Plasmid DNA was isolated by standard techniques and used to transform *S. pyogenes* by electroporation as described previously [29]. Restriction endonucleases, ligases, and polymerases were used according to the recommendations of the manufacturers. GenElute^™^ Bacterial Genomic DNA Kit (Sigma) was used to purify chromosomal DNA of *S. pyogenes*. When required, DNA fragments were purified using Mini Elute™ gel extraction kit (Qiagen) following agarose gel electrophoresis.

### Reverse transcriptase-polymerase chain reaction (RT-PCR)

RNA from *S. pyogenes* cultures was isolated through the Qiagen RNeasy mini kit (Qiagen) combined with mechanical disruption of cells [30]. RNA quality and quantity were measured by comparing the value of absorbance at 260 nm and 280 nm. RNaseOUT™ (RNase inhibitor, Invitrogen) was added to the purified RNA, and the purified RNA was treated with DNase I (Invitrogen) to remove any residual DNA. For RT-PCR, SuperScript™ One-Step RT-PCR with Platinum^®^ Tag (Invitrogen) was used to synthesize cDNA from RNA and to amplify the cDNA by PCR. In order to detect the transcript containing the intergenic region of *gidA* and *gdpP*, *gdpP* and *rpl9*, or *rpl9* and *holB*, the primer sets of *gidA-gdpP*-f (gtaagccagaacttattttgaaacg) and *gidA-gdpP*-r (gatcaaattgaactactccaacagg), *gdpP-rpl9*-f (atcacagaagagatgatgattttcc) and *gdpP-rpl9*-r (atttcacaggaacttcaattagacc), or *rpl9-holB*-f (ctcaagctgaaatattagcagaagc) and *rpl9-holB*-r (agcaattgttacattattccaatcc) were used. These primer sets were supposed to produce about an 1 kb PCR product if an appropriate template exists.

### Creation of a nonpolar inframe deletion mutant

An inframe deletion allele of *gdpP* was created by the method previously described [31]. Briefly, the primers of GdpPInframeEx-f (cgg g a t c cccaacgctgtagaatggtataatcc) and GdpPInframeEx-r (cgg g a t c cctaatacaaagctagcctcaacgtg) were used to amplify an 1.55 kb internal fragment of *gdpP*. (The *Bam*HI restriction sequence in the primers is underlined.) This amplified fragment was inserted into the *Bam*HI restriction site in the pCRII vector (Invitrogen). The resulting plasmid was then used as a template in an ‘inside-out’ PCR reaction with the primers of GdpPInframeIn-f (cca t c g a ttagttgatcatcacagaagagatgatg) and GdpPInframeIn-r (cca t c g a tcaccaaaagaaattcctatgctgag). Cleavage of this fragment with *Cla*I followed by subsequent re-ligation resulted in a nonpolar inframe deletion that replaces DNA sequence encoding E270 – V440 of GdpP with the sequence of gatcgatta encoding DRL. The inframe deletion allele of *gdpP* was then inserted into the *Bam*HI restriction site in the *S. pyogenes*-*E. coli* shuttle vector, pJRS233. The generated plasmid, pJRS233::*gdpP*-IFD, was used to replace the wild type *gdpP* with *gdpP*∆270-440 by a method that employs the temperature sensitivity of the plasmid replication origin as was described previously [31]. Briefly, the plasmid pJRS233::*gdpP*-IFD, was introduced into the wild type HSC5 by electroporation, and transformants were grown in the THY medium containing erythromycin at 30^°^C. Then, the plasmid was inserted into the chromosome through a homologous recombination by shifting temperature to 39^°^C. One colony growing at 39^°^C in the presence of erythromycin was picked and grown at 30^°^C in the THY medium without erythromycin. This temperature shift selects for the excision of the plasmid through another homologous recombination, which could leave either the wild type or deletion allele of the *gdpP* gene on the chromosome depending on the location of the crossover. Since both the wild type revertant and the inframe deletion mutant went through the same process, we used the wild type revertant as the wild type control strain for virulence study using a mouse subcutaneous infection model. The allelic replacement was confirmed by PCR and sequence analysis.

### Construction of a GdpP expression vector and deletion of the transmembrane (TM) domain of GdpP

To express GdpP ectopically, the *gdpP* gene was inserted into an expression vector pABG5, an *E. coli - S. pyogenes* shuttle vector that has the promoter of *rofA* (regulator of fibronectin binding protein A), which has been successfully used to express streptococcal genes previously [32,33]. First, the *gdpP* gene was PCR-amplified with primers of GdpPcomp-f (aaaaaag c a t g cgagtcaatcctgcggacatc) and GdpPcomp-r (aaaaaag g c a c cagcatagcctgttgggacc) and digested with the restriction enzymes, *Sph*I and *Ban*I (Their restriction sequences in the primers are underlined.). The amplified PCR product (~2.2 kb) was then inserted downstream of the *rofA* promoter in pABG5. To create GdpP∆TM, GdpP with the deletion of the transmembrane domain, the GdpP expression plasmid was PCR-amplified with primers of GdpP∆TM-f (gct c t a g acatctttataacctcttaagcacac) and GdpP∆TM-r (gct c t a g acaaaaagaagcttatcaattatc), then the inverse PCR product was digested with *Xba*I and re-ligated (The restriction sequence in the primers is underlined.). The expression of GdpP and GdpP∆TM was confirmed by Western blotting. The expression level of these proteins in various strains was compared to GdpP expression in the wild type by measuring band intensities in digital images of Western blots analyzed with NIH ImageJ software (http://rsbweb.nih.gov/ij/).

### SpeB assay

The proteolytic activity of culture supernatants was quantitated by the method of Hauser et al. [14], which measures the increase in relative fluorescence generated by the proteolytic cleavage of FITC-casein (Sigma). The activity of uninoculated *C* *medium* was used to derive background values. To ensure that all proteolytic activity was specifically the result of SpeB, the cysteine protease-specific inhibitor E-64 (Sigma; final concentration 10 mM) was added to selected samples. This treatment typically reduced activity by >95%. The presence of the pro-protein and processed forms of SpeB was determined through Western blot analysis as described previously [23]. The loading amount of the culture supernatants for Western blotting was normalized based on streptococcal cell numbers as determined by OD_600_. After taken at each time point, the cultures were adjusted to 0.5 of OD_600_ with fresh medium and cells were removed by filtration (0.45 µm of pore size). Then, 10 µl of each culture supernatant was applied to each lane of a SDS-PAGE gel. The amount of the culture supernatants for the proteolytic activity assay was normalized in the same way.

### Murine subcutaneous infection model

The ability of *S. pyogenes* strains to cause disease in soft tissue was evaluated by subcutaneous infection of 6-8 week old SKH1 hairless mice (Charles River Labs) as described previously [34]. Briefly, *S. pyogenes* strains were grown in 10 ml THY media to a mid-logarithmic phase (0.30 of OD_600_), washed, and resuspended in 1ml of sterile saline. This suspension was subjected to brief sonication (3 or 4 pulses of 10 sec each) to disrupt streptococcal chains, and even disruption was confirmed through microscopy. The bacteria were diluted in saline to obtain the appropriate concentration of viable streptococcal cells, which was confirmed by determination of the number of colony forming units (CFU). Then, each mouse received a subcutaneous injection of 10^7^ CFU in a 100 µl volume into the right flank. Each *S. pyogenes* strain was analyzed in a group of 10 mice. The area of the draining ulcers that formed was documented every 24 hrs by digital photography and the precise area contained by each ulcer was calculated from the digital record. Any differences in the areas of ulcers between experimental groups were tested for significance by the Mann–Whitney U-test [35]. The null hypothesis was rejected when *P* values were <0.05. This study was carried out in strict accordance with the recommendations in the Guide for the Care and Use of Laboratory Animals of the National Institutes of Health. This animal study was approved by the Institutional Animal Care and Use Committee (IACUC) of Southern Illinois University (SIU). All mice were anesthetized with isoflurane when the lesion sizes were measured and euthanized by CO_2_ asphyxiation at the end of the experiment.

## Results

### The c-di-AMP phosphodiesterase GdpP was expressed as a multigene transcript in *S. pyogenes*

**Figure 2:**
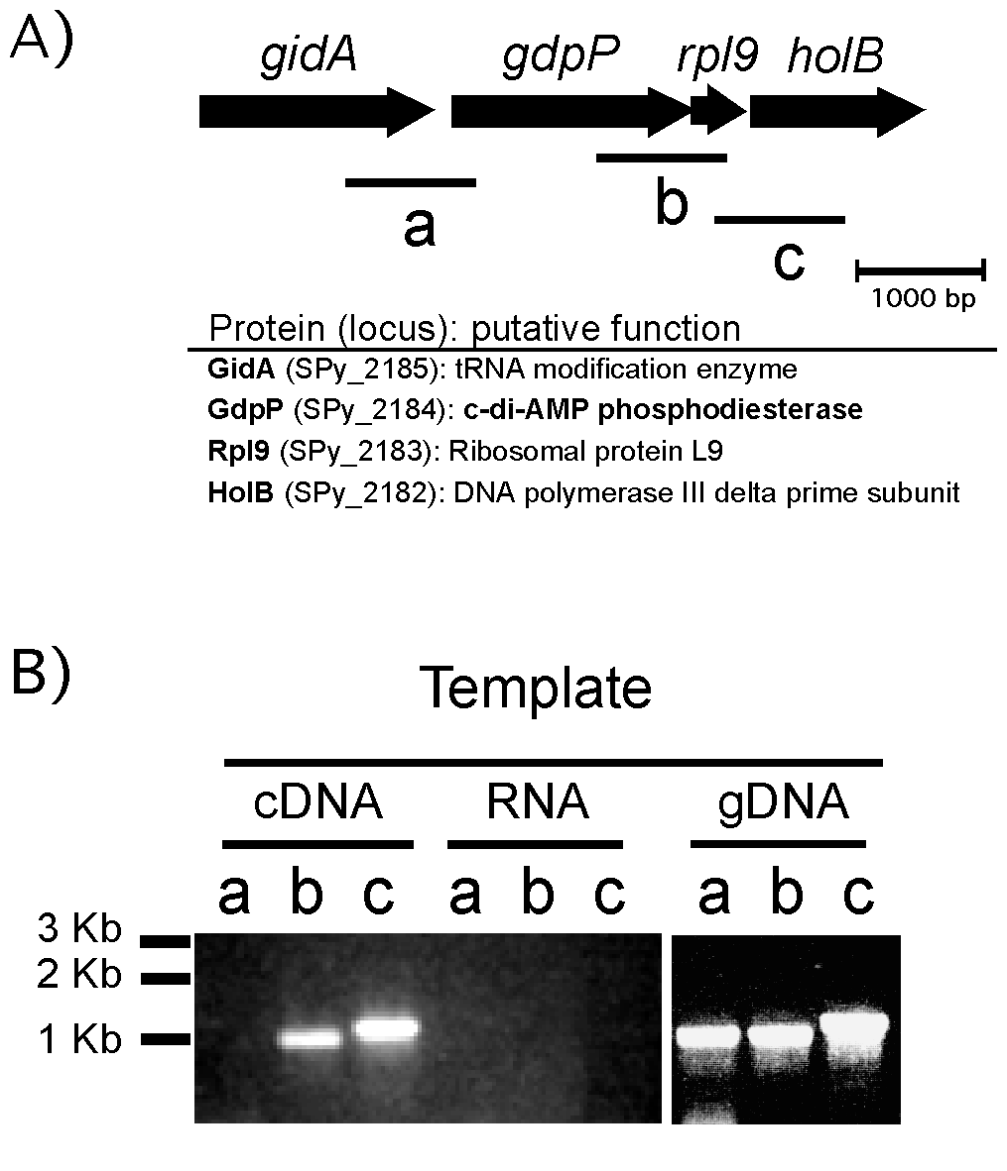
The *gdpP* gene is co-expressed with the downstream genes. A) The organization of the chromosomal region near *gdpP* is shown at the top. The arrows indicate individual open reading frames and their orientations. The predicted proteins encoded by these open reading frames and their putative functions are shown at the bottom. The lines (designated as a, b, and c) under the open reading frames indicate the possible products by the reverse transcriptase PCR performed in this study if an mRNA encompassing those regions is expressed. B) Agarose gel electrophoresis resolving RT-PCR products. RNA extracted from the wild type, HSC5, was converted to complementary DNA (cDNA) with a reverse transcriptase. PCR was then performed using the cDNA as template. Each lane was loaded with a PCR product amplified with each primer set that can amplify the region a, b, or c indicated in A. As a negative control for chromosomal DNA contamination, RNA was used as template, and no PCR product was amplified as expected. As a positive control to confirm that primers work in the PCR reactions, genomic DNA (gDNA) was used as template, and all the PCR products, a, b, and c were amplified.

The *gdpP* gene is located in the middle of genes with the same orientation (Figure 2-A). The gene of GidA, a tRNA modification enzyme involved in the addition of a carboxymethylaminomethyl group to uridine 34 of a subset of tRNAs, is prior to and *rpl9* (ribosomal protein large subunit 9) and *holB* encoding DNA polymerase III delta prime subunit follow *gdpP*. To investigate whether *gdpP* is expressed as a member of a multigene operon or independently, reverse transcriptase-PCR (RT-PCR) was performed (Figure 2-B). Each primer set for RT-PCR was designed to amplify an ~1 kb DNA fragment including the intergenic region between *gidA* and *gdpP*, between *gdpP* and *rpI9*, or between *rpl9* and *holB*. RNA was isolated from the wild type HSC5, and used to prepare cDNA for the template of PCR. In this assay, no PCR amplification occurred between *gidA* and *gdpP*, indicating *gidA* and *gdpP* are not in the same transcript, and this result agrees with the previous one [36]. However, the intergenic regions between *gdpP* and *rpl9*, and between *rpl9* and *holB* were amplified, indicating that *gdpP*, *rpl9* and *holB* are expressed together as one transcript, and the promoter is located in front of *gdpP*.

To delete the *gdpP* gene without affecting the expression of its downstream genes, the internal part of *gdpP* encoding E270 to V440 was deleted without disturbing the frame for translation. The deleted part of GdpP included most of DHH domain, which is responsible for the activity of c-di-AMP degradation (Figure 1). The growth of the *gdpP* deletion strain, ∆GdpP, was not different from the parental strain in both THY and *C* *medium* . The specific growth rates of ∆GdpP and the parental strain were 0.62 hr^-1^± 0.02 (average ± standard deviation) and 0.55 hr^-1^ ± 0.01 in THY medium and 0.64 hr^-1^ ± 0.01 and 0.71 ± 0.01 in *C* *medium* , respectively. In both media, the significance values (*p* values) determined by two-tailed paired Student’s t test between the growth rates of ∆GdpP and the parental strain were greater than 0.05, indicating that their growths were not significantly different.

### GdpP influenced the maturation of the secreted cysteine protease SpeB

**Figure 3:**
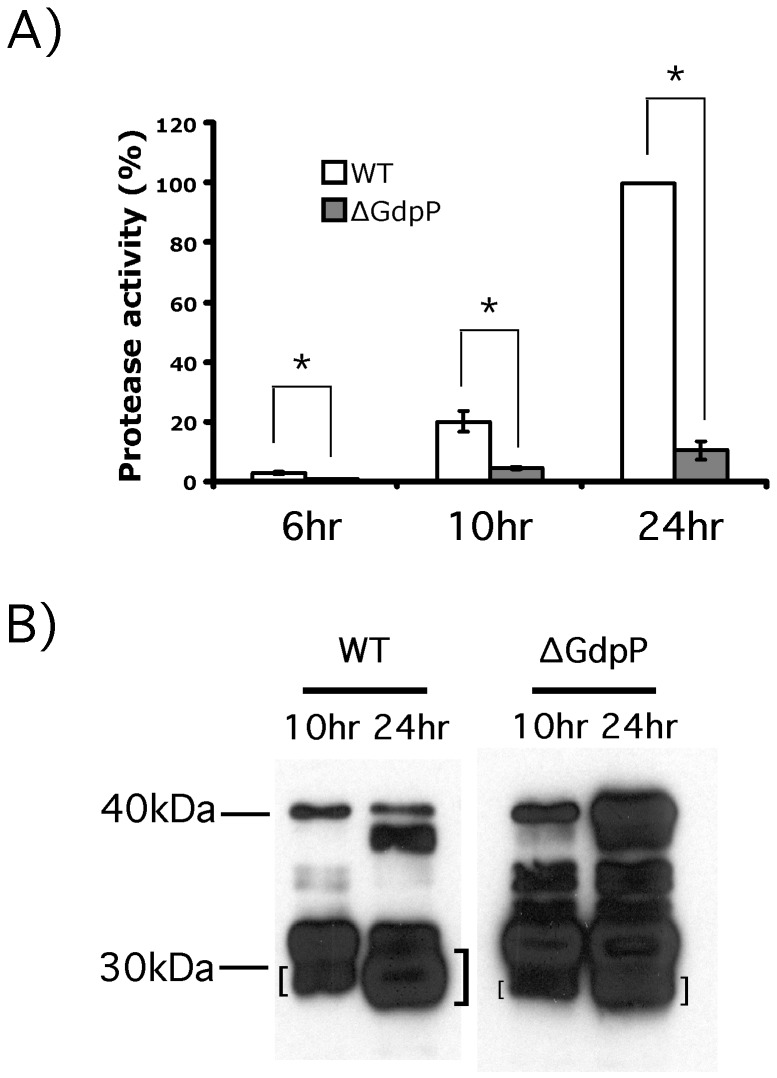
The deletion of *gdpP* impairs the processing of SpeB. A) The protease activity of SpeB secreted from the ∆GdpP strain relative to that from the wild type. The culture supernatants at 6, 10, and 24 hr post-inoculation were taken, and their protease activity was measured and compared to that of the wild type at 24 hr post-inoculation. The protease activity of SpeB secreted from the ∆GdpP strain was much less than that from the wild type. The graph shows the average values and standard deviations for the protease activity assay in triplicate. * indicates significance (p < 0.05) for difference between protease activities of samples as calculated by the two-tailed paired Student’s t test. B) Western blot showing the processing pattern of SpeB. The culture supernatants of *C* *medium* at 10 and 24 hr post-inoculation were taken and used for Western blotting. Secreted 40 kDa proSpeB (the top band of the Western blot) goes through several cleavage steps to become active 28 kDa SpeB (the bottom band of the Western blot marked with "[“or”]"). The SpeB secreted from ∆GdpP has more abundant processing intermediates and less active SpeB. Protein size markers are shown at the left side of the Western blot. The strains used in these assays are: WT, HSC5; and ∆GdpP, the GdpP inframe deletion mutant.

To examine whether or not GdpP is required for the biogenesis of the major secreted protease SpeB, we compared protease activity in *C* *medium* culture supernatant of the *gdpP* deletion strain, ∆GdpP, to that of the wild type strain HSC5. The culture supernatant of ∆GdpP showed much less protease activity of SpeB than that of the wild type (Figure 3-A). The protease activity from ∆GdpP was ~10% of that of the wild type even at 24 hr culture supernatant. Since the transcription rate of SpeB in a *gdpP* knockout strain was the same as that in the wild type [36], we further investigated the effect of GdpP on SpeB biogenesis through Western blotting (Figure 3-B). In the Western blot, although SpeB secretion appeared to be fine, more SpeB processing intermediates and less 28 kDa active SpeB appeared in the ∆GdpP culture supernatant. This result indicates that the processing to produce 28 kDa active SpeB is slower or impaired without GdpP.

### Ectopic expression of GdpP complemented the lower SpeB activity in the ∆GdpP mutant

**Figure 4:**
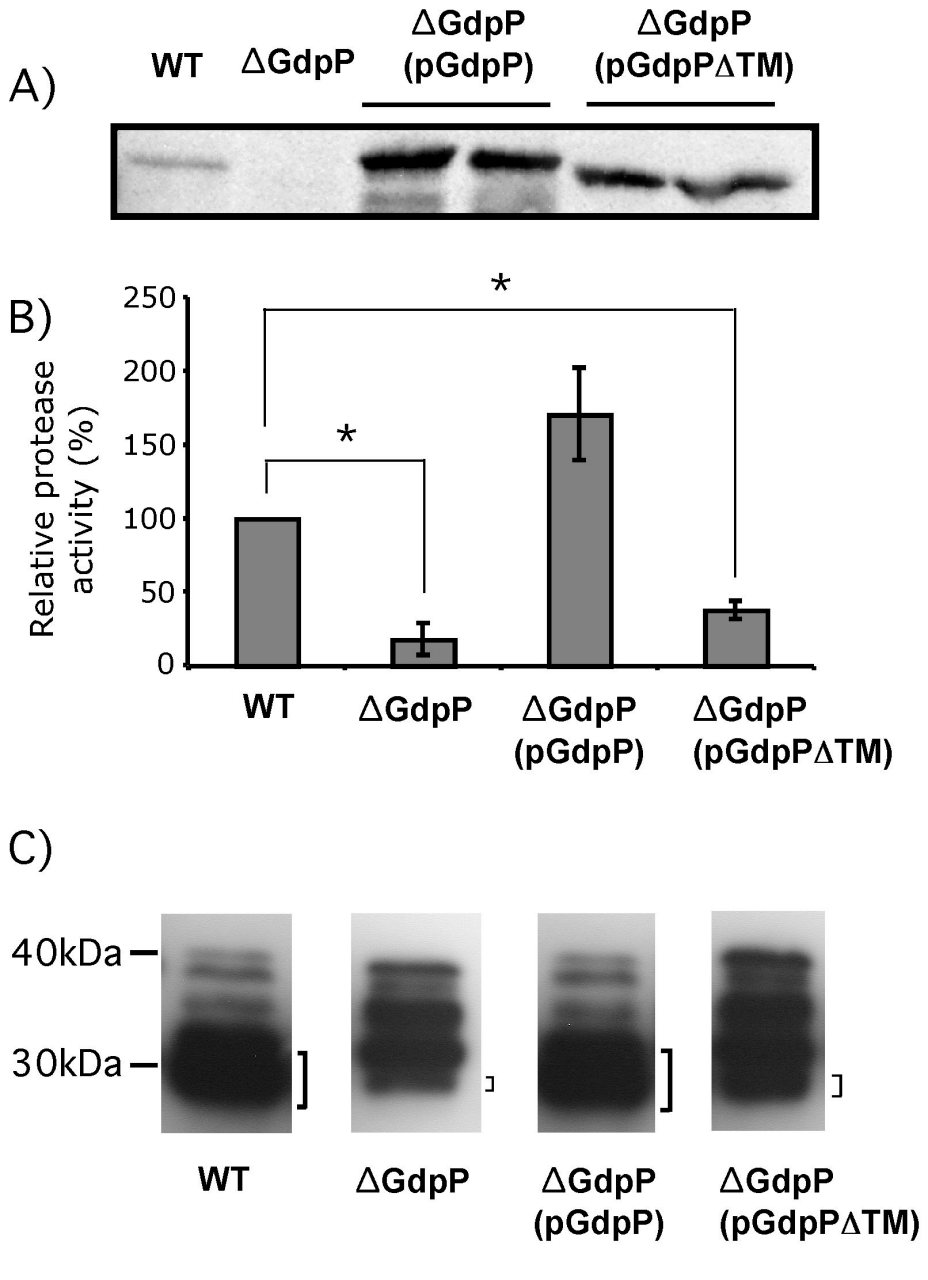
The location of GdpP inside cells is critical for its cellular function. A) A Western blot using a GdpP-specific antiserum. GdpP and transmembrane domain-deleted GdpP (GdpP∆TM) expressed by the *rofA* promoter in a multi-copy plasmid were overexpressed compared to GdpP in the wild type. As expected, the size of GdpP∆TM was slightly smaller than that of GdpP. B) Protease activity of SpeB secreted from strains relative to that from the wild type. Expression of GdpP using a plasmid complemented the lower protease activity of ∆GdpP. However, the expression of transmembrane domain-deleted GdpP (GdpP∆TM) using the same plasmid failed the complementation. The graph shows the average values and standard deviations for the protease activity assay in triplicate. * indicates significance (p < 0.05) for difference between protease activities of samples as calculated by the two-tailed paired Student’s t test. C) Western blot showing the processing pattern of SpeB. Secreted 40 kDa proSpeB goes through several processing steps to become active 28 kDa SpeB (the bottom band of the Western blot marked with"]"). The amounts of active SpeB in the Western blot match with the relative SpeB protease activities of the strains. Protein size markers are shown at the left side of the Western blot. The strains used in these assays are: WT, the wild type HSC5; ∆GdpP, the GdpP inframe deletion strain; ∆GdpP(pGdpP), ∆GdpP expressing GdpP from a plasmid; and ∆GdpP(pGdpP∆TM), ∆GdpP expressing transmembrane domain-deleted GdpP from a plasmid.

We constructed a GdpP expression plasmid and introduced the plasmid into ∆GdpP to examine if this ectopic expression of GdpP can complement the lower SpeB activity in the ∆GdpP strain. To construct the GdpP expression plasmid, the *gdpP* gene was inserted downstream of the *rofA* promoter in the multicopy expression plasmid, pABG5. The *rofA* promoter in the plasmid confers high expression in *S. pyogenes* [32,33]. The GdpP production from the expression plasmid in *S. pyogenes* was confirmed with Western blotting (Figure 4-A). As expected, GdpP was overexpressed (~14.7 fold) from the plasmid compared to that from the chromosomal gene in the wild type. When we determined the protease activity using the culture supernatant of the GdpP-complemented strain, ∆GdpP(pGdpP), the protease activity was comparable to that of the wild type level (~1.7 fold), confirming that the lower protease activity in ∆GdpP was due to the loss of GdpP (Figure 4-B,C).

### The location of GdpP inside cells was critical for its cellular function

GdpP is anchored on the membrane through two membrane spanning helixes at the N-terminus, and this implies that the location of GdpP near the membrane might be critical for its role. To test this hypothesis, we removed the transmembrane domain (Figure 1) from the GdpP expressed from the plasmid and expressed it in the ∆GdpP mutant. As expected, the size of GdpP without the transmembrane domain, GdpP∆TM, was slightly smaller than GdpP and also overexpressed compared to GdpP expressed from the chromosomal gene (Figure 4-A). However, GdpP∆TM did not complement the loss of GdpP in the ∆GdpP strain even though stably expressed; the protease activity and the processing pattern of SpeB from the strain GdpP∆TM was close to those of the ∆GdpP strain (Figure 4-B,C). These results indicate that the transmembrane domain is critical for the cellular function of GdpP.

### The GdpP null mutant exhibited decreased sensitivity to the β-lactam antibiotic ampicillin

**Figure 5:**
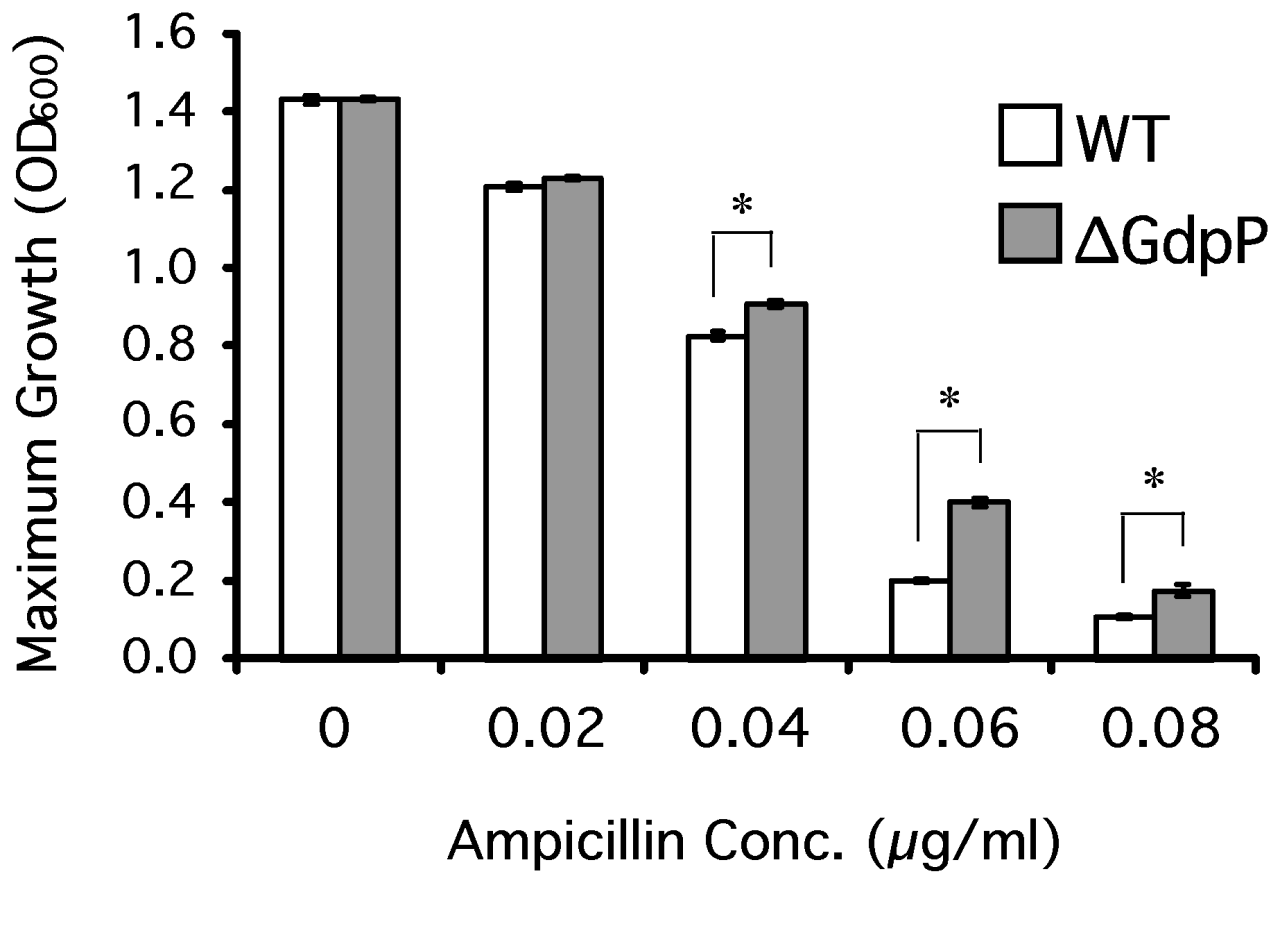
The deletion of *gdpP* in *S. pyogenes* decreases the sensitivity to a β-lactam antibiotic, ampicillin. *S. pyogenes* cells were grown in THY medium containing various concentrations of ampicillin and their maximum growth was measured as the absorbance at 600 nm. The graph shows the average values and standard deviations for each strain’s growth in triplicate. * indicates significance (p < 0.05) for difference between the maximum growths of strains as calculated by the two-tailed paired Student’s t test. The strains used in this assay are: WT, the wild type HSC5; and ∆GdpP, the GdpP inframe deletion mutant.

In *Staph* *aureus* and *B. subtilis*, the *gdpP* deletion decreased sensitivity to cell wall targeting β-lactam antibiotics [5,13]. To test the sensitivity of the GdpP null mutant of *S. pyogenes* to a β-lactam antibiotic, ∆GdpP was grown in THY medium with various concentrations of ampicillin, and maximum growth as the absorbance at 600 nm was measured. As shown in Figure 5, ∆GdpP was less sensitive to ampicillin than the wild type at concentrations between 0.04 µg/ml and 0.08 µg/ml.

### The virulence of ∆GdpP was attenuated in a murine model of soft tissue infection

**Figure 6:**
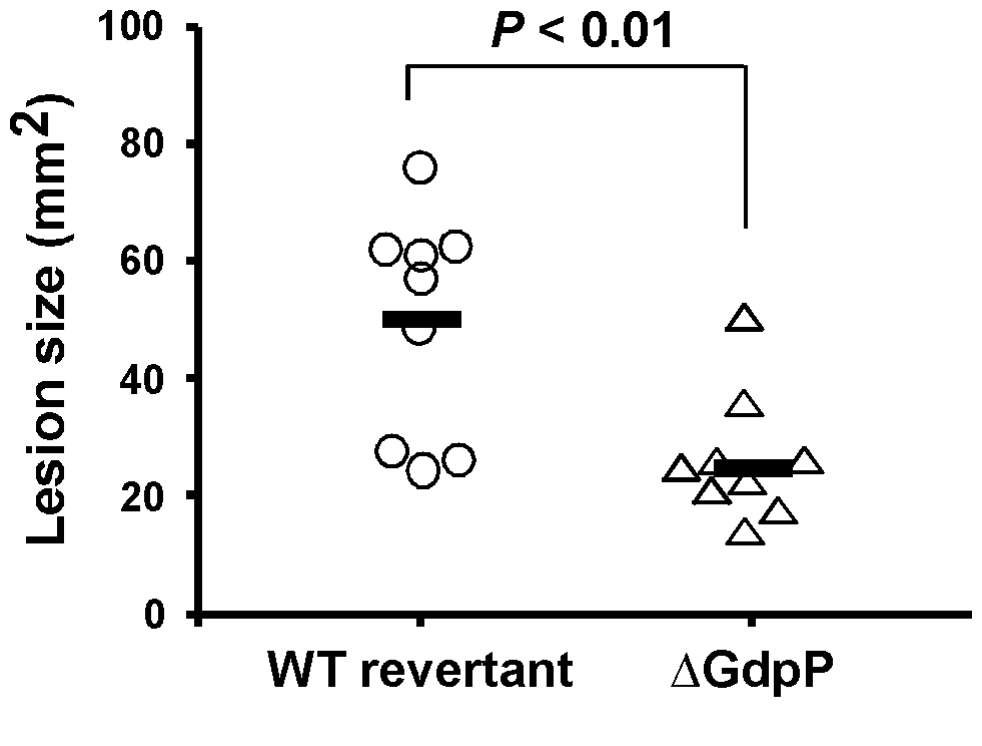
The deletion of *gdpP* attenuates the virulence of *S. pyogenes*. The ability of ∆GdpP to cause disease in the murine subcutaneous infection model is shown. Virulence was evaluated on the basis of the area of the ulcer produced at the time when ulcer formation was maximal (3 days post-infection). The circles and triangles represent ulcer sizes in mice injected with the wild type or the ∆GdpP mutant, respectively. The solid bars indicate the mean values of the ulcer sizes, and *P* value as determined by the Mann–Whitney U test statistic is indicated above the bracket. The following strains were used: WT revertant, the wild type revertant; and ∆GdpP, the GdpP inframe deletion mutant.

The ability of the *gdpP* null mutant (∆GdpP) to cause disease in soft tissue was evaluated using a murine subcutaneous infection model. In this assay, the ulcer size caused by ∆GdpP was compared to that caused by the wild type control strain that is a wild type revertant strain obtained from the inframe deletion procedure. At the last step of inframe deletion, some strains acquire the inframe deletion allele and the others retain the wild type genotype depending on the location of homologous recombination. During the several passages of cultures in the inframe deletion process, we experienced that *S. pyogenes* could loose M protein, a cell surface-anchored major virulence factor that influences virulence in this animal model. Thus, we confirmed M protein expression from both the wild type revertant control and ∆GdpP using Western blotting (data not shown). When we compared the ulcer sizes at 3 days post-infection when ulcer formation was maximal in both strains, ∆GdpP caused smaller lesion sizes than the wild type revertant (Figure 6), indicating that the ∆GdpP mutant was attenuated in its ability to cause lesions.

## Discussion

GdpP is an enzyme hydrolyzing c-di-AMP, which is an emerging secondary messenger in bacteria and archea. The cellular role of this cyclic nucleotide derivative in the pathogenesis of *S. pyogenes* has not been studied yet. The c-di-AMP synthesis enzyme, di-adenylate cyclase, appears to be essential for survival in many pathogens [5,7,9–11]. However, the c-di-AMP phosphodiesterase, GdpP, is not essential, so the *gdpP* gene has been deleted in several Gram-positive bacteria to examine its cellular role. In this study, we created a *gdpP* deletion strain of *S. pyogenes* and examined the role of GdpP in the biogenesis of SpeB, the sensitivity to the cell wall targeting antibiotic ampicillin, and virulence.

Our RT-PCR revealed that the *gdpP* gene is expressed with at least two other downstream genes, *rpl9* and *holB*. The *rpl9* gene is the gene of ribosomal protein L9. Hoffman et al. determined the structure of Rpl9 of *Bacillus stearothermophilus* ribosomes through X-ray crystallography and NMR spectroscopy [37]. The ribosomal protein is a highly elongated protein containing two domains separated by a nine-turn connecting helix. In the crystal structure of *Deinococcus* *radiodurance* ribosomes [38], the location of Rpl9 is between the large subunit and the small subunit of the ribosome. Some studies showed that Rpl9 might be involved in translational fidelity. The study by Herr et al. on translational bypassing of bacteriophage T4 gene 60 in *E. coli* suggests that Rpl9 defects stimulate ribosome slippage by enhancing mRNA movement through the ribosome [39]. The next gene *holB* is the gene of a DNA polymerase III delta prime subunit. Even though *holB* is essential for growth in *E. coli* [40], a *S. pyogenes holB* kockout strain was viable (unpublished data). We do not know the biological significance of the coexpression of these genes, but the activity of GdpP may influence the fidelity of replication through HolB or of translation through Rpl9.

There are several ways that a SpeB zymogen is converted to active SpeB. A zymogen can cleave the pro region by itself in a reduced condition [41,42]. However, this intra-molecular autocatalysis is much slower than the inter-molecular autocatalysis in which an active SpeB activates the other zymogens by cleaving the pro region [42]. Thus, most processing seems to occur by inter-molecular autocatalytic processes. At least six intermediates form during the processing to active SpeB [42]. Previous studies show that GdpP appears to be involved in cell wall homeostasis. The deletion of the *gdpP* gene in *Staph* *aureus* and *B. subtilis* increases the degree of peptidoglycan cross-linking and resistance to beta-lactam antibiotics [4,5]. The deletion of *gdpP* gene in *S. pyogenes* also increased the resistance to the beta-lactam antibiotic, ampicillin, suggesting that GdpP in *S. pyogenes* could also influence the cross-linking status of the cell wall. Most secreted proteins in *S. pyogenes* are exported through the general secretory (sec) pathway. SpeB is secreted through Exportal, which is a general sec system forming in a distinct cellular membrane microdomain [43] containing enriched anionic phospholipids to facilitate protein translocation [44]. HtrA, a serine protease, is located in Exportal and accelerates the speed of SpeB processing [25,26], so the perturbed cell wall synthesis or assembly caused by *gdpP* disruption might disturb the formation of Exportal or mislocate HtrA, which could influence SpeB processing in turn. Another possibility is that the increased amount of c-di-AMP due to the absence of GdpP might influence the biogenesis of RopA or PrsA. Both proteins are peptidyl-prolyl *cis-*trans isomerases (PPIase) required for the full conversion of proSpeB to active SpeB [22–24]. The PPIase enzymes catalyse the cis/trans isomerization of peptidyl–prolyl bonds in nascent polypeptides, which is often a rate-limiting step in protein folding [45].

GdpP is a membrane-anchored protein. It has a non-cleavable signal sequence containing two membrane-spanning helixes at the N-terminus. Thus, GdpP hydrolyzes c-di-AMP near the cell membrane. The multi-copy expression of *gdpP* in the chromosomal *gdpP* deletion mutant restored SpeB activity to more than the wild type level (Figure 4). However, when the transmembrane domain-deleted GdpP was expressed, SpeB activity was not complemented any more (Figure 4). This result indicates that the location of GdpP inside cells is important for at least SpeB biogenesis. If SpeB processing is influenced by the defect of cell wall homeostasis, then c-di-AMP concentration near the cell wall could be important for cell wall homeostasis.

**Figure 7:**
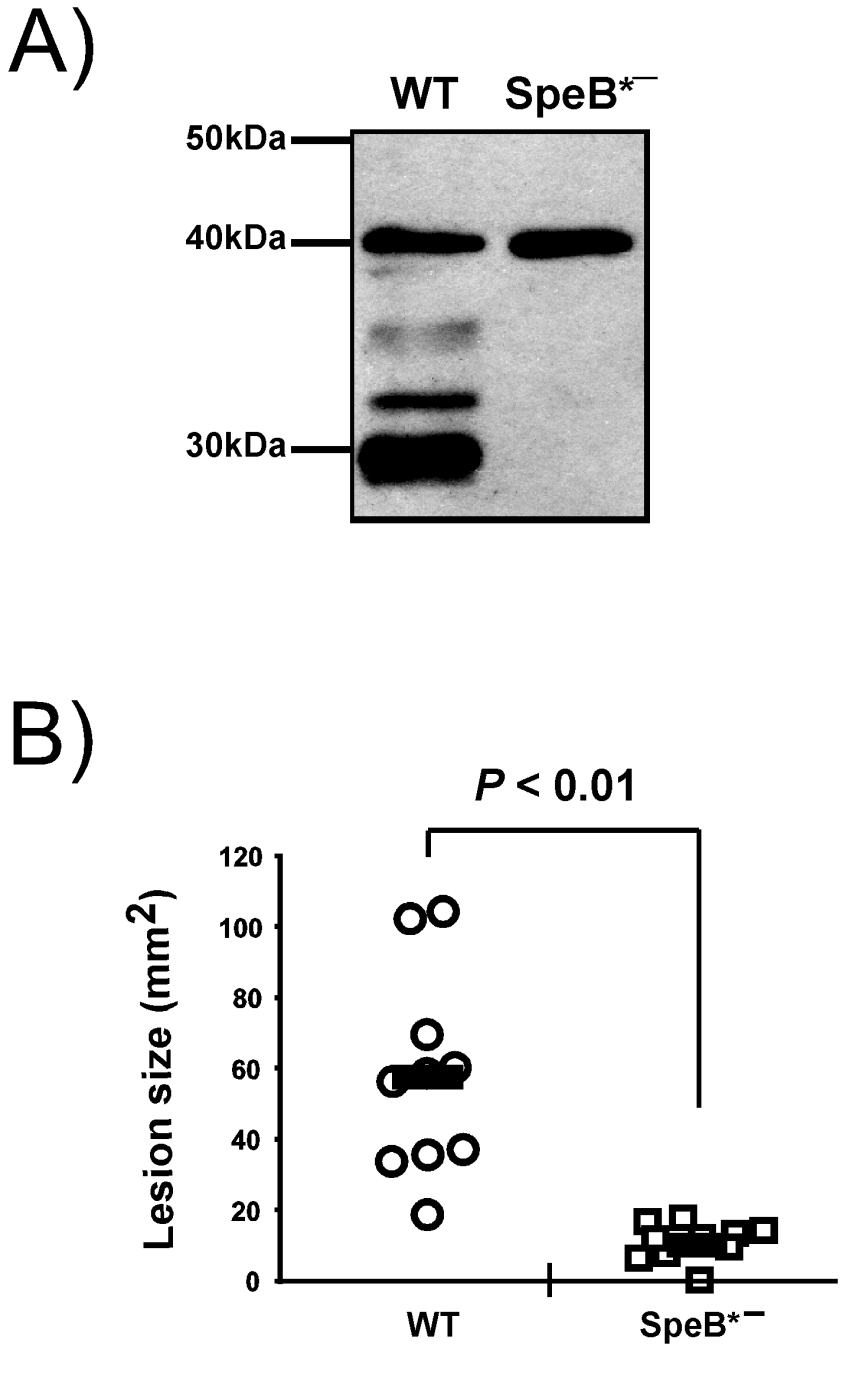
Removal of protease activity from SpeB attenuates the virulence of *S. pyogenes*. A) Western blot showing the processing pattern of SpeB. The culture supernatants of *C* *medium* at 24 hr post-inoculation were taken and used for Western blotting. Secreted 40 kDa proSpeB from the wild type goes through several processing steps to become active 28 kDa SpeB. The SpeB secreted from the protease activity-negative SpeB mutant, SpeB*^−^, however, does not go through any processing. Protein size markers are shown at the left side of the Western blot. B) The ability of the SpeB*^−^ strain to cause disease in the murine subcutaneous infection model. Virulence was evaluated on the basis of the area of the ulcer produced at the time when ulcer formation was maximal (3 days post-infection). The circles and squares represent ulcer sizes in mice injected with the wild type or the SpeB*^−^ strain, respectively. The solid bars indicate the mean values of the ulcer sizes, and *P* value as determined by the Mann–Whitney U test statistic is indicated above the bracket. The following strains were used: WT, the wild type HSC5; and SpeB*^−^, the protease activity-negative SpeB mutant JWR10.

The deletion of the *gdpP* gene attenuates the virulence of *S. pyogenes* in a murine model of subcutaneous infection. Probably, the processing defect of SpeB in the ∆GdpP contributes to the attenuation since removing protease activity from SpeB in HSC5 attenuates virulence in the same infection model. The strain JWR10 is a HSC5 mutant in which an amino acid residue in the active site of SpeB, cysteine 192, is replaced with serine [46]. This mutation abolished protease activity of SpeB, so no SpeB processing occurred from the mutant SpeB (Figure 7-A). When the virulence of JWR10 was examined with the same animal model system, virulence was more attenuated than that of ∆GdpP (Figure 7-B). However, we cannot exclude other possibilities such that the attenuated virulence of ∆GdpP could be due to at least in part disturbed expression of other virulence factors or disturbed cell wall homeostasis.

## Conclusion

In this study, we demonstrated that the deletion of the *gdpP* gene in the human pathogen *S. pyogenes* impairs the processing of SpeB, decreases sensitivity to the beta-lactam antibiotic ampicillin, and attenuates virulence in a murine model of subcutaneous infection, indicating that GdpP has an important role in maintaining *S. pyogenes* virulence.
